# Human Anatomy

**Published:** 1899-01

**Authors:** 


HUMAN ANATOMY. A Complete, Systematic Treatise by Various Authors. Including a Special Section on Surgical and Topographical Anatomy. Edited by Henry Morris, M. A., M. B., Lond.; Senior Surgeon to the Middlesex Hospital; Examiner in Surgery in the University of London; Member of the Council and Chairman of the Court of Examiners of the Royal College of Surgeons of England; Honorary Member of the Medical Society of the County of New York. Illustrated by 790 wood cuts, the greater part of which are original and made expressly for this work by special artists, over 200 of which are printed in colors Second edition, revised and enlarged. Royal 8vo, pp. xxx.—1274. Philadelphia: P. Blakiston's Son & Co. 1898.

To a certain proportion of students anatomy has been and probably will continue to be a dry, difficult and somewhat uninteresting study. It being an exact science, a collection of facts, the merit of each individual work on the subject, as well as the excellence of each teacher will depend largely upon the presentation of the matter in such way as to overcome as much as possible the features stated.

To render the study interesting the bare facts should be as much as possible associated with the indications wherein they will be of future service and application. To render it less difficult, the items of almost mere knowledge should be carefully pruned or minimised and the important points and their application coincidently emphasised.

Knowledge which cannot be applied is very much like money which cannot be spent. This fact applies to anatomy probably more than any other branch of medicine.

Henry Morris in presenting the anatomy known under his name, has certainly not lost sight of the requirements indicated, which are of so much importance. In his work, he has selected as colaborers for their special fitness such men as J. Bland Sutton, Frederick Treves and others, all of whom serve as an additional guarantee of the character of the volume.

The illustrations and descriptions connected with them are beyond criticism, and in this work they are made quickly intelligible by the use of contrasting different type in designating the various structures. Arteries, veins, lymphatics, are all in contrasting colors, and in the section on osteology the origin and insertion of muscles are outlined on the various bones in different or contrasting colors, conveying at a glance a mental picture of the gross features and action of the particular muscle, in a particularly happy way. Whether due to the ingenuity displayed in type, simplicity with accuracy of design, or color, the illustrations in this work appear not only attractive but quickly instructive and particularly and noticeably so.

Especially satisfactory also is the topographical anatomy and illustrative drawings—many of them new. To any one interested in anatomy or desirous of being so interested this book cannot but be attractive, for it more than ordinarily fulfills its mission. It can be recommended without reservation of any kind, but for students, its clearness, simplicity, comprehensiveness and illustrations make it more than acceptable. If a student cannot learn anatomy and how to use anatomical knowledge with the aid of this work, he cannot learn it at all.

The bookmaker has also done his part, in fact all who have had to do with it, have without question been qualified and fulfilled their obligation. So far as observable, errors are not in evidence.

The volume will doubtless be a much used text-book, combining as it does, brevity with excellence. W. H. H.

